# Tremor in Multiple Sclerosis—An Overview and Future Perspectives

**DOI:** 10.3390/brainsci10100722

**Published:** 2020-10-12

**Authors:** Karim Makhoul, Rechdi Ahdab, Naji Riachi, Moussa A. Chalah, Samar S. Ayache

**Affiliations:** 1 Neurology Division, Lebanese American University Medical Center Rizk Hospital, Beirut 113288, Lebanon; karim.makhoul@lau.edu (K.M.); rechdi.ahdab@lau.edu.lb (R.A.); Naji.riachi@lau.edu.lb (N.R.); 2 Gilbert and Rose Mary Chagoury School of Medicine, Lebanese American University, Byblos 4504, Lebanon; 3 Hamidy Medical Center, Tripoli 1300, Lebanon; 4 Service de Physiologie-Explorations Fonctionnelles, Henri Mondor Hospital, AP-HP, 94010 Créteil, France; moussachalah@gmail.com; 5 EA 4391, Excitabilité Nerveuse et Thérapeutique, Université Paris-Est-Créteil, 94010 Créteil, France

**Keywords:** tremor, multiple sclerosis, movement disorder, cerebellum, basal ganglia, thalamus, cerebello-thalamo-cortical pathways, pseudo-tremor

## Abstract

Tremor is an important and common symptom in patients with multiple sclerosis (MS). It constituted one of the three core features of MS triad described by Charcot in the last century. Tremor could have a drastic impact on patients’ quality of life. This paper provides an overview of tremor in MS and future perspectives with a particular emphasis on its epidemiology (prevalence: 25–58%), clinical characteristics (i.e., large amplitude 2.5–7 Hz predominantly postural or intention tremor vs. exaggerated physiological tremor vs. pseudo-rhythmic activity arising from cerebellar dysfunction vs. psychogenic tremor), pathophysiological mechanisms (potential implication of cerebellum, cerebello-thalamo-cortical pathways, basal ganglia, and brainstem), assessment modalities (e.g., tremor rating scales, Stewart–Holmes maneuver, visual tracking, digitized spirography and accelerometric techniques, accelerometry–electromyography coupling), and therapeutic options (i.e., including pharmacological agents, botulinum toxin A injections; deep brain stimulation or thalamotomy reserved for severe, disabling, or pharmaco-resistant tremors). Some suggestions are provided to help overcome the unmet needs and guide future therapeutic and diagnostic studies in this complex disorder.

## 1. Introduction

Multiple sclerosis (MS) is the leading cause of nontraumatic neurological disability in young adults [1]. Tremor has long been recognized as an important symptom of the disease, Charcot himself having included tremor in the disease defining triad along with nystagmus and scanning speech [2]. Tremor as a symptom has a negative impact on the quality of life (QoL) in general, let alone MS patients with other often disabling neurological symptoms [3]. In the setting of MS, a considerable number of subjects report unemployment and disability with increasing tremor severity [4,5], and up to one third are compelled to modify their daily activity to cope with this debilitating symptom. Therefore, proper characterization of tremor in MS and recognition of its subtypes is crucial to developing a treatment plan tailored to the patients’ need and help reduce its impact on the QoL. We hereby summarize our current understanding of MS related tremor and the unmet needs and future direction in this complex disorder.

## 2. Epidemiological Background

Epidemiological studies suggest that the prevalence of tremor in MS is somewhere between 25% and 58% [3,5,6]. These variable estimates are possibly the result of referral bias; whereby specialized MS centers tend to recruit more severely affected patients [6]. Alternatively, the tool used to assess tremor could significantly influence its estimated prevalence and explain these discrepancies. As compared to clinical scales, accelerometric methods are more sensitive by including subjects with asymptomatic (clinically undetectable) tremor which may occur in up to 20% of patients [5]. Perhaps the most accurate figure is that proposed by Rinker et al. based on the NARCOMS patient population, which is the largest to date to have studied MS tremor (more than 14,000 patients) [4]. Some 45–46.8% were found to have tremor based on a self-filled clinical scale. Since this study did not use accelerometric methods, patients with asymptomatic tremor would have been overlooked. On the other hand, these results better reflect the true clinical impact of MS tremor by excluding those unlikely to experience any related functional impairment. The major limitation of these studies is their cross-section design. A precise estimate of the frequency and impact of tremor is only possible using a cohort design with longitudinal tremor evaluation using valid and reliable tremor assessment tools. Only then will the natural course of this symptom and its relation to disease activity be properly defined.

## 3. Clinical Manifestations

MS characteristically causes a large amplitude 2.5–7 Hz [7] predominantly postural or intention tremor [6] both of which classified under the umbrella term ‘action tremor’ in the consensus of tremor established by the movement disorders society [8]. Although one would expect intention tremor to be the more prevalent subtype of tremor in MS based on the lesional concept of the disease and in reference to Charcot triad [2], this is not supported by available data. Alusi et al. found that postural tremor was significantly more common than its intentional counterpart (44% versus 6%, respectively). Although Parkinsonism has been reported in MS [9], isolated resting tremor has been rarely described. Recently, Ayache et al. employed a thorough neurophysiological assessment of MS tremor, using accelerometric techniques coupled with electromyographic (EMG) monitoring and reported that in the majority of cases, MS patients displayed a pseudo-rhythmic activity rather than true tremor [10]. In their study, among 32 MS patients selected with hand clumsiness attributed to a postural tremor according to the description of Alusi et al. [5], only one was found to have a genuine action tremor, reflected by a rhythmic EMG and accelerometric activity (4 Hz in frequency). The remaining had rather a type of pseudo-rhythmic activity attributable to continuous adjustment–readjustment of movements due to cerebellar dysfunction [10]; such an activity would clinically mimic a tremor and could be referred to as a ‘pseudo-tremor’.

Common body parts involved in MS tremor are the upper and lower extremities, head [11,12,13], trunk [4] and vocal cords [14]. The tongue and jaws are not classically affected [15]. Palatal tremor (also known as palatal myoclonus) has been described in isolated case reports [16,17,18,19]. The latter may occur in combination with pendular nystagmus in an entity known as ‘oculopalatal tremor’, characterized by rhythmic movements of the soft palate produced by contractions of the tensor or levator veli palatine. The encountered 2–3 Hz frequency makes oculopalatal tremor closer to a myorrhythmic activity and is typically present in a context of brainstem insult [20].

Correlation of tremor with MS disease severity has not been formally studied and can only be inferred from data derived from the available epidemiological studies. Rinker et al. used Patient Determined Disease Steps (PDDS) score to mark MS disease severity in their cohort of MS patients with tremor [4]. Although not widely used, this score showed high correlation with Expanded Disability Status Scale (EDSS) [21] and the huge advantage of not requiring direct physician contact and examination which makes it a good tool for surveys. Using this approach, the correlation was low with Archimedes’ spiral drawing, and therefore there was no evidence to support that disease severity was correlated with tremor. On the other hand, Alusi et al. were able to demonstrate a significant correlation between tremor and disease severity as measured by EDSS, nine-hole peg test and Barthel index of activities of daily living [5]. Similarly, tremor scores in Pittock et al. study were highly correlated with disease severity as measured by EDSS [22]. The latter results seem to suggest that tremor is one of those progressive symptoms that worsen along with the disease course.4. Pathophysiological Mechanisms.

The pathophysiology of tremor remains incompletely understood. MS tremor has been classically attributed to lesions in the cerebellum or cerebellar peduncles or cerebello-thalamo-cortical pathways based on its co-occurrence with other signs and symptoms such as dysarthria, dysmetria, dysdiadochokinesia, and dystonia [5]. For instance, several clinical studies suggested a correlation between upper limb dystonia symptoms and MS tremor, supporting the idea of the involvement of cerebello-pallido-thalamo-cortical network dysfunction in the pathophysiology of MS tremor [23]. Moreover, neuroimaging (tractography) data obtained from patients who underwent deep brain stimulation (DBS) for tremor, including those with MS, highlighted the dentato-rubro-thalamic tract as the common stimulation target for DBS [24]. Recently, structural and functional magnetic resonance imaging studies revealed the involvement of cerebello-thalamo-cortical pathways in the generation of MS tremor [25,26]. In these studies, MS patients with tremor exhibited cerebellar and thalamic atrophy, increased cerebellar lesion load ipsilateral to the tremor side, and hyperactivation of several areas (ipsilateral inferior parietal lobule, premotor and supplementary motor areas) during the performance of a motor task, compared to those without tremor [26]. In addition, significant correlations were found between tremor severity and thalamic atrophy/lesion load and superior cerebellar peduncle atrophy, but not with whole brain lesion load [25]. The involvement of the cortical and pontine components of this network has been also highlighted by several studies. For example, a case series suggests the implication of inflammatory demyelinating cortical peri-central sulcus lesions in the generation of intention tremor in MS, despite the absence of rubro-cerebellar lesions [27]. In another study assessing the relationship between MS tremor and infratentorial lesion load, a significant correlation was found between tremor amplitude and the lesion load in the contralateral pons, or in the bilateral pons when considering patients with severe tremor [28].

In addition to the abovementioned neuroimaging works, the implication of the cerebello-thalamo-cortical pathway in the pathogenesis of MS tremor has been also supported by some DBS studies. The latter postulated that thalamic ventralis intermedius nucleus (VIM) DBS restored cerebello-cortical inhibition usually imposed on the primary motor cortex in normal subjects and involved in maintaining movements [29,30]. This point was proven valid through studying transcranial magnetic stimulation induced cerebellar inhibition during the on and off phase stimulation periods of DBS. Ayache et al. recognized that cerebellar inhibition was absent in the off phase stimulation in comparison to the on phase stimulation providing further support to the involvement of the cerebello-thalamo-cortical fibers in the pathophysiology of tremor [30].

Despite all the above described data, cerebellar involvement in MS tremor has never been unequivocally proven [31,32]. The positive response of selected cases to DBS of Ventralis Oralis Posterior (VOP) (the basal ganglia output) and VIM of the thalamus seems to suggest a role of the basal ganglia [5]. The rarity of rest tremor and the lack of response to anti-parkinsonian medications argue against such an involvement. In addition, the responders’ rate to DBS is only 60–70%, which suggest that other structures might be involved in the generation of tremor.

In addition to the basal ganglia and cerebellar pathways, some particularities have been found in specific cases of MS tremor. For instance, using a visually cued simple reaction time task, some authors have linked action tremor to a pathological oscillator at a supra-spinal level [33]. Moreover, in specific types of tremor (i.e., palatal tremor), the symptom severity has been correlated with a Guillain–Mollaret triangle lesion as noted in some MS cases [16,17,18,19].

To conclude, MS tremor seems to be best explained by a multilevel injury rather than a specific culprit area. In other words, MS tremor seems to result from multiple disconnections within the cortico-cerebello-subcortical networks instigated from pathologies documented in these areas in patients suffering from MS.

## 4. Assessment Modalities

The difficulty in assessing MS tremor resides in the variability of tremor types the clinician is confronted with. Several clinical and neurophysiological methods have been implied in the assessment of MS tremor ranging from common tasks employed in the neurological exam (e.g., finger-to-nose task, spiral drawing, handwriting) [34] to visual tracking [35,36,37], accelerometric techniques, accelerometry–EMG coupling [10], Stewart–Holmes maneuver [38], and digitized spirography [39,40]. In addition, some tremor rating scales have been studied in MS. Alusi et al. validated the Bain Score for Tremor Severity (BSTS) in this population [34]. Marrie et al. validated the Tremor and Coordination Scale (TACS) in the NARCOMS registry [41]. Hooper at al. worked on the Fahn Tremor Rating Scale (FTRS) [42]. Daudrich et al. employed the multidimensional assessment of tremor (MAT) in MS [43]. Despite the validity and practicality of those utilized tools, there is no unified rating scale or method being implicated among MS patients in general as a routine assessment tool by global MS societies in order to combine available literature and synthesize a general evidence-based concept about tremor in MS. Even though it is possible to project certain implications from available epidemiological studies through associating pieces of evidence, there remains a clear lag in terms of unification of tremor assessment.

Still it is difficult to decide on a unified validated scale to endorse as a standardized testing method, since the validation methods were variable and the information provided by the scores themselves target different aspects of tremor. For example, the TACS score is a simple six-option patient-oriented score [41]. Although easy to use, the score might be lacking important information. The FTRS, a more complex scale, involves assessment of all body parts that might be affected by tremor in addition to spirography and functionality as reported by patients [42]. The scale, however, when implemented in MS revealed high intrarater reliability but some interrater variability [42] which makes it difficult to rely on when patients are examined by several physicians. It is important to note that the authors omitted the patient-based functionality part in the reliability study, as it was not applicable by their methods. The authors as well did not validate the score with established assessments.

In light of such a difficulty in tremor assessment, perhaps the most useful tools remain to be those dependent on neurophysiological studies. However, despite their objectivity and low interrater variability, accelerometric techniques make it difficult to differentiate movements due to ataxia from movements due to tremor per se. For this sake, Carpinella et al. were interested in validating an electrophysiological method coupling the accelerometer to a gyrometer in which analysis was able to control for other movements, and the method isolated intention tremor from other components [44]. Not only are such techniques important for assessment and characterization of tremor but are valid as well to differentiate tremor types and implement treatment as suited.

In a study by Ayache et al., MS patients cohorted for hand clumsiness with or without visible tremor were assessed with an accelerometer paralleled to EMG assessment. In regard to neurophysiological techniques of accelerometry coupled to EMG; the phenomenon manifested by a visual MS tremor could be rather attributed to a pseudo-rhythmic activity as only one patient among the 32 patients in the studied cohort exhibited a true tremor reflected by a perfect accelerometric and EMG coupling [10]. In this case, future MS tremor epidemiologic studies would benefit from neurophysiological assessments to offer a clearer view as to the prevalence of this entity and its evolution along the course of the disease.

The same team used the Hilbert Hung transform to further analyze the data (EMG and accelerometric data) obtained in MS patients with visible tremor and compared them to those obtained in a group of healthy subjects. The results were notable for the existence of some similarities between patients tremor and physiological tremor detected in the healthy group [45]. With such a finding, neurophysiological techniques, if well implemented, might improve tremor detection in MS and help further stratify patients to appropriate therapeutic approaches.

Another study that confirmed the utility of neurophysiological assessment techniques in selecting patients for specialized treatment approaches was that performed by Liu et al. In this study, the patients were asked to perform a tracking task and a spectral analysis of tremor frequency was analyzed. Patients thereafter underwent thalamotomy. The latter was found to be more efficient in patients with a tremor characterized by a single frequency peak in comparison to those having a multiple frequency tremor [37].

## 5. Pharmacological and Alternative Treatments

In terms of pharmacological management of tremor in MS, evidence has been inconclusive regarding a definite therapeutic approach. Several medications have been stated as effective throughout literature; however, solid evidence was contradictory with studies providing good results and others failing to obtain such an effect.

Isoniazid, for example, has been used in the 1980s, and was the subject of mostly open-label studies [46,47,48,49] and few randomized placebo-controlled cross-over trials [50,51]. From a mechanistic point of view, some authors suggested that isoniazid effect on tremor occurs via its inhibitory action of monoamine oxidase [51], while others suggested the possibility of GABAergic modulation in small but specific brain areas [50]. Mild or marginal response was documented when using conventional or higher doses. In one of the controlled trials, no significant changes were observed on objective measures (i.e., tremogram) despite the subjective clinical improvement [50]. The current limited evidence in conjunction with the rarity and the small size of the available works, and the important side-effect profile (appearance of lower limb weakness and increase of spasticity in [49]) do not support the use of this medication for the management of MS tremor.

Levetiracetam, an antiepileptic drug with a favorable pharmacological profile, has been considered as well for MS tremor. Its binding site (i.e., the synaptic vesicle protein SV2A) is found in high concentration in several brain areas (e.g., dentate gyrus, superior colliculus, thalamic nuclei, cerebellum, cerebral cortex) [52]. In addition, levetiracetam effects include partial inhibition of N-type voltage channels and the zinc mediation of GABA responses [15]. In the context of MS tremor, levetiracetam might act by decreasing the action of cortico-ponto-cerebellar pathways or restricting the high-frequency repetitive firing of neuronal cells [52]. Despite some trials providing promising evidence for its use, others were not in favor of this effect. However, it remains to be established whether the specific profiles of patients might have influenced the results. Open-label studies provided promising benefits for this drug in the context of MS tremor. For instance, Striano et al. documented an improvement in tremor activity on a daily living questionnaire and tremor index in MS tremor patients [52]. Similarly, Chitsaz et al. found levetiracetam to be effective in reducing tremor as measured by the Tremor Group Rating Scale [53]. However, the available placebo-controlled crossover trials yielded inconsistent results. While Solaro et al. established the effective improvement of smooth trajectory by tremor patients using neurophysiological assessment, the clinical assessment measures used did not improve in a statistically significant manner [54]. The same team recently documented a significant reduction in tremor questionnaire in patients who received levetiracetam followed by placebo intervention (but not vice versa) [55]. Finally, Fey at al. did not observe any significant effect on clinical scores, functional assessment, and spirography [56]. The discrepancy in the available literature could be partly explained by the study design, the sample size, the heterogeneity of the recruited cohorts (different disease phenotypes and tremor types), and tremor assessment tools (different clinical and physiological outcomes).

Topiramate is another antiepileptic drug that might act on tremor via its antagonistic activity on a subtype of glutamate receptors, which counteracts hyperexcitability and increases GABAergic activity [57]. Although topiramate revealed some promising results in a case report [57], the evidence presented in a supporting small open-label trial involved patients with tremor etiologies other than MS making it difficult to generalize the effect from a heterogeneous population [58].

Cannabis was tried to manage persistent symptoms of MS such as tremor and spasticity. The anti-tremor effect of cannabis could involve the cholinergic, GABAergic, serotonergic, beta-adrenergic, and cannabinoid systems [59,60]. Cannabis seemed to be beneficial for spasticity in MS. However, when tested for tremor, positive results were documented in a case report [61], but placebo-controlled trial resulted in mild [59] or no improvement [62,63,64]. In addition to the inconclusive effect of cannabinoid use in MS tremor, a detrimental side effect profile has been described regarding cannabis use. Many psychiatric features have been also associated with the drug including anxiety, panic, mood, and psychotic disorders in susceptible individuals. The drug might result in cognitive deficits in high doses [65]. Moreover, autonomic dysregulation manifesting as tachycardia, dizziness, orthostatic hypotension, and dry mouth was well recognized [66].

Ondansetron, a 5-hydroxytrypatmine receptor antagonist might be helpful in the context of tremor by acting on cerebellar serotoninergic system [67]. Although an initial placebo-controlled trial has suggested potential effects of this agent on tremor [68], a more recent open-label study failed to replicate the findings [69]. However, the results of the first trial should be interpreted with caution since the cohort was not only constituted of MS patients but also contained patients suffering from cerebellar tremor of different etiologies [68].

Furthermore, it is worth mentioning that very few case reports and case series documented tremor improvement following primidone (i.e., barbiturate anticonvulsant) [70,71], 4-aminopyridine (i.e., potassium channel blocker) [72], glutethimide (i.e., piperidinedione derivative with sedative-hypnotic and anticholinergic effects) [73], and ethanol [74].

Apart from these pharmacological interventions, some authors were interested to target MS tremor by modulating the underlying autoimmune process of MS itself. In a comparative study by Rinker et al., a significant improvement in Tremor Related Activities of Daily Living score was noted among patients treated with natalizumab (humanized monoclonal antibody) compared to those taking other disease modifying drugs [75]. Based on these results, immune modulation using natalizumab may constitute an appealing treatment for MS tremor, by limiting lymphocyte trafficking into the central nervous system; however, the results of this trial should be interpreted with caution because of its retrospective design, and future prospective randomized controlled trials would help drawing formal conclusion regarding the potential role of natalizumab, and other immunomodulatory/immunosuppressive treatments in this clinical context.

In addition to oral drugs, some authors were interested in assessing the effects of botulinum toxin A injection on MS tremor. A pilot study did not establish any statistical significance [76]; however, a double-blind controlled trial revealed significant findings and improvement on the BSTS, writing, and Archimedes spiral drawing [77]. The authors hypothesized that botulinum toxin effects on MS tremor might have occurred by blocking muscle spindle afferents and gamma motor efferents. While several variable factors play a role in orally administered pharmacological therapy besides the drug dosing, botulinum toxin A injection procedures are dependent on the approach followed by the physician. They have to be phenomenally driven in terms of understanding the tremor mechanisms in each patient and thus targeting the muscles involved in the movement as such. This could probably explain the discrepancy between the two studies. In the first study, the toxin was injected into the flexor and extensor compartments of the most severely affected forearm with a total of two injections only, without EMG guidance. In the second study, the patients received injections based on the tremor pattern observed in conjunction with EMG guidance. Despite the described clinical effectiveness of botulinum toxin A in tremor and besides injection site irritations incurred by the patient, there is no doubt that this procedure bears some other disadvantages [78,79]. To start, transient local muscle weakness could occur and may be avoided/minimized by starting at a lower dosing during the first session of injection and titrating as needed at the hands of an experienced clinician. Another important complication to mention is the development of neutralizing antibodies subsequent to recurrent injections. Such a resistance could be overcome by the use of botulinum toxin B or a different botulinum toxin A molecule with less immunogenicity [78]. It is also worth mentioning systemic botulism which has been very rarely reported as a side effect [79].

A summary of the pharmacological interventions is provided in Table A1.

With failure of conceptualizing an appropriate MS tremor treatment algorithm based on isolated pharmacological approach, physicians might be oriented to seek surgical therapies bearing in mind the heavy complications that might be faced. The most favorable approach up to the moment was the DBS approach. Despite being surgical, the method is adjustable based on the patient’s response. Again, DBS does not result in complete tremor resolution. It might be as well cumbersome to the patient in the long run due to frequent reprogramming and not encompassing a sustainable effect [80]. The patient must be referred to such procedures only when labeled by a severe or disabling tremor, tremor refractory to medical therapy, not affected by sensory or motor deficits limiting functionality in the studied limb, stable disease before surgery, and relatively preserved cognition [80].

There have been several studies providing evidence towards the use of DBS in MS tremor, but the definite type of tremor with an indication to use DBS has not been clearly investigated, and the target location to place the electrodes has not been well established. Thirteen patients studied by Geny et al. had tremor improvement up to 69% with DBS of the VIM nucleus as assessed by tremor amplitude and functional scales [81]. The improvement of the tremor was more noticeable in the proximal components rather than the distal ones. Nandi and Aziz revealed a better improvement at 6 months in postural tremor in comparison to intention tremor (up to 63% vs. 36% improvement in tremor frequency (compared to pre-procedural values)) [82]. They suggested that distal single frequency hand tremor is best targeted by VIM nucleus or VOP nucleus of the thalamus, while a mixed proximal-distal tremor with a broader frequency range would most likely to benefit from a combination of VOP–ZI (zona incerta) stimulation. Probably triaging the patients as per the study of Nandi and Aziz could have further improved the distal tremor component that was noticed to have a less responsive effect in the Geny et al. trial. Another study by Schulder et al. performed on nine patients revealed significant decrease in clinical tremor scores [83]. Berk et al. established comparable values of tremor reduction with statistically significant results. This was the only study that provided insight into the overall QoL improvement [84]. Counterintuitively, no statistical significance was attained concerning QoL improvement. Whether this is related to the MS disease progression or not needs to be investigated through further studies.

Apart from DBS, a more invasive approach has been proposed for the management of MS tremor. In fact, thalamotomy has been adopted by several teams worldwide and was mainly reserved for severe intention upper limb MS tremor [85]. Technically, this procedure consists of partial ablation of the thalamus using either a direct surgical intervention (guided or not by MRI or computed tomography) or a less invasive method by applying radiofrequency (i.e., gamma knife thalamotomy). Efficacy of such an intervention varied across studies, with some describing an improvement over the first year after the surgery, followed by a recurrence of the symptoms [85]. In addition, outcome measures showed a great heterogeneity across the published experiences, sample sizes were small, and several works reported on serious postoperative complications. All these elements render the judgement of the utility of this intervention a difficult if not an impossible task.

To summarize, regarding tremor treatment in MS, one could initiate a pharmacological agent based on each patient profile in conjunction with the clinician’s solid experience. Several studies are warranted in the field to address the possible effectiveness of each agent with stratification of the patients based on tremor type. In case of failure of a pharmacological therapy, the most plausible next step is to refer the patient to a specialized and experienced center with the ability to inject botulinum toxin A based on tremor phenomenology. The last resort for a resistant tremor would thus be DBS in specialized center that can target the appropriate part of the thalamus to stimulate.

## 6. Conclusions and Perspectives

It is no wonder that the efforts described in literature to address MS tremor are crucial and developmental; however, it is important to mention that at this stage of available evidence, there must be a pathway to orient future studies probably by leaving the mission for world-renowned MS societies to excavate any gaps in the topic. Still, the provided studies clarified many aspects that help the physicians to push their own clinical sense towards the management of MS tremor. It is, thus, definite, that one third to a half of MS patients might develop tremor during their disease course, mostly postural and intention type tremor. MS tremor could correspond to a genuine low frequency tremor and be related to a neural generator located somewhere between the brainstem and the cerebellum. In addition, tremor observed in this population might have its roots in an exaggerated physiological tremor, or it might reflect a cerebellar dysfunction, in such a way that it would be related to a pseudo-rhythmic activity due to continuous adjustment and readjustment of a limb position. Finally, given the psychological burden of the disease, some MS patients would suffer from a psychogenic tremor, the latter being rarely if never thoroughly assessed in the literature. Although MS tremor can be assessed through several validated scales, neurophysiological techniques are highly needed in order to differentiate among the four previously mentioned MS tremor types and carefully adapt the therapeutic approach. Treatment remains open to the physicians to practice their art of clinical sense in terms of providing pharmacological therapy, or referring the patient to a specialized botulinum or DBS center.

## Appendix A

**Table A1:** Summary of pharmacological trials that targeted tremor in multiple sclerosis (MS).

Medication	Studies	Study Design	Main Results
Isoniazid	Sabra et al. [46]	Open-label case series (four MS patients)	Improvement in the studied patients
	Duquette et al. [47]	Open-label trial (13 MS patients)	Mild improvement in 10 patients on at least one of the assessment methods
	Morrow et al. [48]	Open-label case series (five MS patients)	Improvement in four patients
	Francis et al. [49]	Open-label pilot trial (five MS patients)	No improvement with conventional dose; marginal clinical improvement with dose increase 2–3 fold reduction in tremor on goniometry
	Bozek et al. [50]	Double-blind, placebo-controlled, crossover trial (10 MS patients)	Clinical improvement in six out of eight patients who completed the study No significant change on tremograms results Better response in postural vs. intention tremor
	Hallet et al. [51]	Double-blind, placebo-controlled, crossover trial (six MS patients)	Improvement in all patients on at least one of the assessment methods
Levetiracetam	Striano et al. [52]	Open-label trial (14 MS patients)	Improvement in 11 patients who completed the study
	Chitsaz et al. [53]	Open-label trial (22 MS patients)	Transient improvement in 20 patients who completed the study
	Solaro et al. [54]	Double-blind, placebo-controlled Crossover trial (eight MS patients)	Significance changes in kinematic but not clinical measures in the six patients who completed the study
	Solaro et al. [55]	Double-blind, placebo-controlled, crossover study (48 MS patients)	Improvement in the subjective but not kinematic measures in patients who received levetiracetam followed by placebo intervention (but not vice versa)
	Fey et al. [56]	Double-blind, placebo-controlled, crossover trial (18 MS patients)	No improvement in the 14 patients who completed the study
Topiramate	Schroeder et al. [57]	Case report	Clinical improvement
	Sechi et al. [58]	Open-label trial (nine patients with cerebellar tremor, of which five had MS)	Clinical and neurophysiological improvement Treatment discontinuation in three patients prior to trial completion
Cannabis	Clifford [59]	Single-blind, placebo-controlled trial (eight MS patients)	Mild subjective but not objective improvement in 5/8 patients; subjective and objective improvement in two patients
	Meinck et al. [61]	Case report	Clinical and neurophysiological improvement
	Wade et al. [62]	Double-blind, placebo-controlled study (160 MS patients)	No subjective changes in tremor (154 patients including 13 patients with tremor as primary symptom)
	Zajicek et al. [63]	Multicenter placebo-controlled trial (spasticity as primary outcome) (630 MS patients)	No clinical improvement
	Fox et al. [64]	Double-blind, placebo-controlled crossover trial (14 MS patients)	No clinical and neurophysiological improvement
Ondansetron	Rice et al. [68]	Double blind, placebo-controlled crossover study in 20 patients (16 MS patients; three patients with cerebellar degeneration, one patient with lithium intoxication)	Clinical improvement
	Gbadamosi et al. [69]	Open-label pilot study (14 MS patients)	No clinical improvement
Primidone	Henkin & Herishanu [70]	Case series (two MS patients)	Improvement in two patients
	Naderi et al. [71]	Open-label pilot study (10 MS patients)	Clinical improvement
4-aminopyridine	Schniepp et al. [72]	Case report	Clinical improvement
Glutethimide	Aisen et al. [73]	Open-label study (six MS patients, two patients with traumatic brain injury)	Functional benefits in six out of eight patients
Natalizumab	Rinker et al. [75]	Comparative retrospective trial (natalizumab vs. other disease modifying drugs) (567 MS patients)	Clinical improvement in natalizumab-treated patients
Botulinum toxin A injection	Clarke et al. [76]	Open-label pilot study (five MS patients)	No clinical improvement
	Van der Walt [77]	Double-blind, controlled crossover trial (23 MS patients)	Clinical improvement
